# Adolescents' Sexual Health During the COVID‐19 Outbreak: A Systematic Review

**DOI:** 10.1002/hsr2.70774

**Published:** 2025-04-29

**Authors:** Elahe Ahmadnia, Arezoo Haseli, Atefeh Davoudian, Mina Abbasi

**Affiliations:** ^1^ Department of Midwifery School of Nursing and Midwifery, Zanjan University of Medical Sciences Zanjan Iran; ^2^ Family Health and Population Growth Research Center, Health Policy and Promotion Research Institute Kermanshah University of Medical Sciences Kermanshah Iran; ^3^ Deputy of Research and Technology Zanjan University of Medical Sciences Zanjan Iran

**Keywords:** 2019 nCoV infection, SARS coronavirus 2 infection, sexuality, teenager, youths

## Abstract

**Background and Aims:**

The COVID‐19 pandemic has greatly disrupted adolescents' access to sexual health services, resulting in a decline in their overall sexual well‐being. This systematic review explored adolescent sexual health during the COVID‐19 pandemic.

**Methods:**

A systematic review of quantitative studies—including observational research, clinical trials, and quasi‐experimental interventions—examined English‐language articles published between January 2020 and February 10, 2025, sourced from databases such as PubMed, Web of Science, Scopus, and Google Scholar. Study quality was evaluated using the Newcastle−Ottawa Scale (NOS) for observational studies, Cochrane RoB 2 for clinical trials, and ROBINS‐1 for quasi‐experimental designs. Due to the heterogeneity of the data.

**Results:**

After identifying 781 articles, 10 studies with a total sample size of 636,873 participants were included in the final systematic review. Observational studies during the COVID‐19 pandemic revealed diminished access to sexual and reproductive health (SRH) services, greater dependence on informal information sources, widening health inequalities, and negative impacts on adolescent sexual behavior. Intervention studies on online SRH education demonstrate significant positive impacts across key areas: access to SRH services, safe sex practices, communication with parents about sexual health, lower acceptance of dating violence, normative beliefs regarding adolescent sexuality, HIV/STI awareness, and condom use. The findings emphasize notable improvements in communication, knowledge, and attitudes toward sexual health, driven by these targeted interventions.

**Conclusion:**

The COVID‐19 pandemic disrupted adolescent SRH globally, reducing service access and amplifying inequities. While some behaviors (e.g., sexual activity) showed resilience, systemic gaps in education and healthcare persist. Multisectoral efforts are needed to ensure adolescents' SRH rights are upheld during crises. However, the interventional studies underscore the viability of digital, media‐literate interventions in improving adolescent sexual health.

**Trial Registration:**

The review study was officially registered on the PROSPERO website on 02/08/2023 under the code CRD42023438631 and received approval from the jury.

## 1 Introduction

The COVID‐19 pandemic has greatly impacted global well‐being, as noted by the World Health Organization (WHO) [1]. By August 18, 2021, there were about 208 million confirmed cases and 4.37 million deaths worldwide [2]. This situation has increased anxiety, stress, fears of infection and job loss, reduced physical activity, and altered sleep patterns [2, 3]. It has also led to social distancing and unemployment, significantly altering lifestyles. Many have experienced loss and separation from loved ones, decreased healthcare seeking, and shifts in sexual behaviors, which could result in issues like sexual dysfunction and unintended pregnancies, which negatively affect quality of life [3, 4].

The COVID‐19 pandemic has disrupted daily life and access to essential health services [5], posing unique challenges for adolescents in maintaining sexual health [6]. Sexual health is vital for overall well‐being, influencing physical, psychological, and social development [7]. As public health authorities adapt, understanding effective interventions for adolescents is critical [8, 9]. Research highlights that adolescents are particularly affected by changes in sexual health, which plays a key role in fostering healthy relationships and human development [10, 11]. The COVID‐19 pandemic may have significantly impacted their sexual well‐being [10, 12].

Adolescent pregnancy poses risks such as increased intimate partner violence, depression, and suicide [13]. It often results in pregnancy‐related complications, which are a leading cause of death among females aged 15–19. Additionally, children of adolescent mothers face higher risks of prematurity, low birth weight, and infant mortality [14, 15, 16]. The limitations on social interaction caused by home confinement and school closures have limited adolescents' social interactions, affecting sexual behaviors, relationships, contraception access, protection against sexually transmitted infections (STIs), and overall health. Social distancing has led to reduced sexual activity, fewer partners, lower sexual desire, and changes in pornography use [17, 18]. Promoting sexual health is vital to prevent STIs and unintended pregnancies [19].

Educating adolescents on responsible sexual behavior is vital, with school sex education playing a key role [20]. School‐based interventions have been shown to improve sexual health [21]. Educating adolescents about responsible sexual behavior is crucial, and sex education in schools plays a key role in addressing these concerns [20]. Effective school‐based interventions, including policy changes and community engagement, enhance sexual health [21]. However, the COVID‐19 pandemic has made web‐based interventions increasingly important [22]. UNESCO recommends utilizing digital health resources for their accessibility, privacy, and engaging content [23]. Research in Thailand shows that online education can effectively prevent adolescent pregnancies [24]. Additionally, health literacy significantly influences sexual health, empowering individuals to understand health information and make informed decisions [25].

Despite numerous studies examining adolescent sexual health before and during the pandemic (Widman et al. 2019 [26, 27, 28, 29]), many existing studies have primarily focused on traditional face‐to‐face interventions. These methods have become less feasible due to social distancing and quarantine measures (He et al., 2020). Most reviews on the impact of COVID‐19 on sexual and reproductive health (SRH) focus on young adults and older individuals, making their findings less applicable to adolescents aged 10–19 [22, 30, 31, 32]. Another systematic review explored SRH in adolescents, but it was not conducted during the pandemic [29, 33]. In contrast, our study specifically examines SRH in adolescents aged 10–19 during the COVID‐19 outbreak. This systematic review aims to address the urgent need for evidence on innovative approaches used to promote adolescent sexual health in this context. By combining the findings of recent studies, we seek to provide a clearer understanding of what works, what does not, and why.

The primary clinical question guiding this review is: What has been the impact of COVID‐19 on adolescent sexual health, and how effective have interventions been in enhancing sexual health outcomes for adolescents during the pandemic? This study is particularly relevant as it can inform future public health strategies and clinical interventions aimed at improving sexual health outcomes for adolescents in similar crises [34]. The findings of this study contribute to a deeper understanding of how guidelines can be implemented to address the needs of this vulnerable population during times of widespread disruption.

This study provides novel insights by reviewing various types of quantitative research methods, including observational studies, randomized controlled trials (RCTs), and quasi‐experimental interventions conducted during the pandemic. We focus on digital platforms, telehealth, and community‐based initiatives to evaluate adolescent sexual health during COVID‐19. The novelty lies in our examination of how these interventions influence sexual activity, awareness of sexual violence, and knowledge of HIV and STIs among adolescents. Additionally, the review highlights the limitations of past studies, which often lack robust methodologies or fail to capture the complex effects of the pandemic on adolescent behavior.

In conclusion, this study explores the assessment and impact of pandemic measures on adolescents' sexual activities and relationships, highlighting potential long‐term effects. It emphasizes the need for adaptable sexual health interventions during global crises like COVID‐19. Through a systematic review, the research addresses gaps in the literature and proposes strategies to enhance adolescent sexual health services during current and future public health emergencies. Therefore, we conducted a systematic review to examine adolescent sexual health during the COVID‐19 outbreak.

## 2 Materials and Methods

### 2.1 Eligibility Criteria, Information Sources, and Search Strategy

Study eligibility for this systematic review was assessed by two authors (M.A. and E.A.), based on the following inclusion criteria:

1. Peer‐reviewed articles published between January 2020 and February 10, 2025.
2. Articles written in English.
3. Quantitative studies, including observational research, RCTs, and quasi‐experimental interventions.
4. Studies on expectant adolescents should target healthy individuals aged 10−19, as defined by WHO [35].
5. Studies examining the SRH of adolescents who have natural sexual orientations and tendencies.

This systematic review has been registered on PROSPERO (ID: CRD 42023438631) and adheres to the Preferred Reporting Items for Systematic Reviews and Meta‐Analyses (PRISMA) guidelines [36]. The research was structured using the PICO framework:

Participants (P): Adolescents aged 10–19.

Intervention (I): Online sexual and reproductive health education.

Outcomes (O): Sexual and reproductive health activities.

Study design (S): RCTs or quasi‐experimental studies.

Comprehensive searches were conducted across multiple databases, including Web of Science, Scopus, PubMed, and Google Scholar, using an advanced search strategy. Boolean operators (AND and OR) were utilized to refine the results, and search terms were tailored to suit the requirements of each database. Standardized keywords were sourced from the MeSH browser, with a focus on terms such as “adolescents,” “sexual health,” and “COVID‐19.” The search encompassed studies published between January 2020 and February 10, 2025, with no geographical limitations (see Appendix SA).

Exclusion criteria included studies that did not align with our objectives, as well as reviews, books, conference abstracts, theses, editorials, unpublished data, case series/reports, qualitative studies, and articles with overlapping results.

### 2.2 Study Selection

The studies that met the reviewers' eligibility criteria were selected for a full text review. Two researchers (E.A. and M.A.) independently screened the abstracts of all identified studies to determine their relevance. Subsequently both researchers evaluated the full texts of the studies to decide on inclusion. Any disagreements between the researchers were resolved through discussion. If consensus could not be reached, a third reviewer was consulted to make the final decision.

### 2.3 Data Extraction

Two authors (M.A. and E.A.) independently extracted and screened eligible studies and removed duplicates. They then cross‐checked the data in a blinded fashion to ensure accuracy. Discrepancies were resolved through discussion or by consulting a third reviewer. Data were systematically extracted using a structured form that included significant details such as the first author's name, publication year, country of origin, and study type. Additionally, important sample characteristics were noted, including source population, sample size, age, results, and the Newcastle−Ottawa Scale (NOS) [37] and Cochrane Risk of Bias 2 (RoB2) [38] are used for quality assessment. For quasi‐experimental studies, the ROBINS‐I tool was used [39]. Characteristics of the studies included in the systematic review are presented in Table 1.

**Table 1:** Characteristics of individual studies included in the review.

First author's name/year (reference)	Type of study	Setting/location	Age Range of participants (years)	Sample size		Gender		Intervention's type	Study outcomes	Findings in intervention group versus control group	Quality assessment
				Intervention group	Control group	Female	Male				
Anastario et al. [40]	RCT^a^	Fort Peck Reservation in Montana/United State	15−18	96	22	NR	NR^b^	School‐based SRH curriculum/during 17 weeks	Sexual activity	More sexual activity (IRR = 3.6)^c^	Low^j^
Scull et al. [41]	RCT	School/United State	14.42 ± 0.70^d^	216	374	52.70%	42.99%	Media literacy education for sexual health promotion/4−5 sessions	‐Dating violence, ‐Normative beliefs, ‐Communication patterns	‐Less acceptance of dating violence in boys versus girls (1.44 ± 0.05 vs. 1.60 ± 0.70). ‐More interaction of gender for normative beliefs about teen sex (*p* < 0.05) ‐More sexual health communication with a parent (*p* < 0.05)	Some concerns^j^
Nelson et al. [42]	Pilot RCT	Social media sites^e^/United State	16.0 ± 0.9	77	77	NR	NR	Community‑informed, online HIV prevention intervention/uses nine modules	‐Knowledge of HIV/STIs ‐Knowledge of pornography	‐High HIV/STI knowledge scores (13.70 ± 2.6 vs. 12.8 ± 3.4) ‐More knowledge of pornography (75%)	Low^j^
Thongnopakun et al. [43]	Quasi‐experimental research	Public schools in the province of the East region/Thailand	15–19	37	34	54.1% of IG	47.1% of CG	“Our love, our control” online program on SHL^f^/8 weeks	‐Sexual health literacy ‐Behaviors to prevent unintended pregnancies and STIs	‐More sexual health literacy score (MD^g^: 11.20; 95% CI: 3.79−18.61). ‐More scores of behaviors about preventing unintended pregnancy and STIs (MD: 23.92; 95% CI: 16.56−31.29)	Some concerns^k^
Yount et al. [44]	RCT	27 wards were selected of the city/Nepal	12–16	Group 1: 379 Group 2: 358	387	99%	1%	The CARE's TPP^h^/NR	‐SRH^i^ knowledge	More sexual and reproductive health knowledge (*p* = 0.036)	Low^j^
Hong et al. [45]	Cohort longitudinal	United States	13–18	Total = 371 participants, Mal	—	—	100%	—	Condomless anal sex, HIV testing, STI testing, and rate of PrEP use	The rate of condomless anal sex declined from 23.2% to 9.7%, not significantly (OR = 0.89, 95% CI [0.69−1.16]). HIV testing among all males significantly decreased during the pandemic (OR = 0.78, 95% CI [0.61−0.99], *p* = 0.041). STI testing among males stayed stable at 19.6%−20.3%, with a change of 0.6% (95% CI [−2.8−4.0]). PrEP use fell from 2.1% to 0.0% during the pandemic, with no significant difference noted (OR = 0.94, 95% CI [0.56−1.60])	7^l^
Vandermorris et al. [46]	Cross‐sectional	Canada	12–19	Total = *n* > 630,000 female	—	100%	—	—	Pregnancies contraception, sexually transmitted infections	The rate of adolescent pregnancies during the pandemic was 1.12 (95% CI: 1.02–1.23) Contraception rates during the pandemic were 9.2 per 1000 adolescent females, lower than the expected 11.3 (rate ratio 0.82; 95% CI: 0.77–0.89). Sexually transmitted infections: 1.2 versus 2.2 per 1000 adolescent females (rate ratio 0.52; 95% CI: 0.51–0.53)	8^l^
Bonett et al. [47]	Cross‐sectional	31‐clinic, hospital/United States	15–21	Total = 2770	—	NR	NR	—	STI testing	STI tests declined for chlamydia (28%), gonorrhea (29%), syphilis (19%), and HIV (19%). However, positivity rates rose for chlamydia (10.4%−12.7%, *p* = 0.003) and gonorrhea (1.7%–3.4%, *p* < 0.001), with no significant changes for syphilis or HIV	9^l^
Alamolhoda et al. [48]	Cross‐sectional	High schools/IRAN	13–19	Total = 1300 Male	—	—	100%	—	Sexual health	Sexual health score: 50.35 (± 9.05)	9^l^
Montalti et al. [49]	Cross‐sectional	Metropolitan City of Bologna, Italy	14–19	Total = 378	—	40%	61%	—	Sexual and reproductive health	58.2% of adolescents reported that the COVID‐19 pandemic negatively affected their relationships and sexual lives	7^l^

^a^ Randomize control trail.

^b^ Not reported.

^c^ Incidence rate ratio.

^d^ Mean ± standard deviation.

^e^ Facebook and Instagram.

^f^ Sexual health literacy.

^g^ Mean difference.

^h^ Tipping point program.

^i^ Sexual and reproductive health.

^j^ Quality assessment (Rob‐2).

^k^ ROBINS‐1 tool.

^l^ Quality assessment (NOS).

### 2.4 Assessment of Risk of Bias

The risk of bias was independently assessed by two authors (E.A. and M.A.) using the Cochrane Risk of Bias 2 (RoB2) tool for randomized trials [37]. This tool assesses studies concerning sample selection bias (randomization process), execution bias (deviation from the intended intervention), sample drop in results (missing outcome data), outcome measurement bias, and reporting of results (selection of the report outcome). Studies were categorized as having a low risk, some concerns, or a high risk of bias. Disagreements between reviewers were resolved through discussion, and a consensus was reached (see Appendix SB1). Risk of bias visualization was performed using the online Robvis tool [50]. For observational studies, the NOS was used to assess quality [37]. Articles scoring 7 or higher are considered high‐quality, those scoring between 5 and 6 are deemed average‐quality, and those scoring 4 or lower are classified as low‐quality (see Appendix SB2).

### 2.5 Data Synthesis

Due to the heterogeneity of the included data, a meta‐analysis was not feasible. Therefore, a qualitative synthesis of the study results was conducted.

## 3 Results

### 3.1 Study Selection

**Figure 1:**
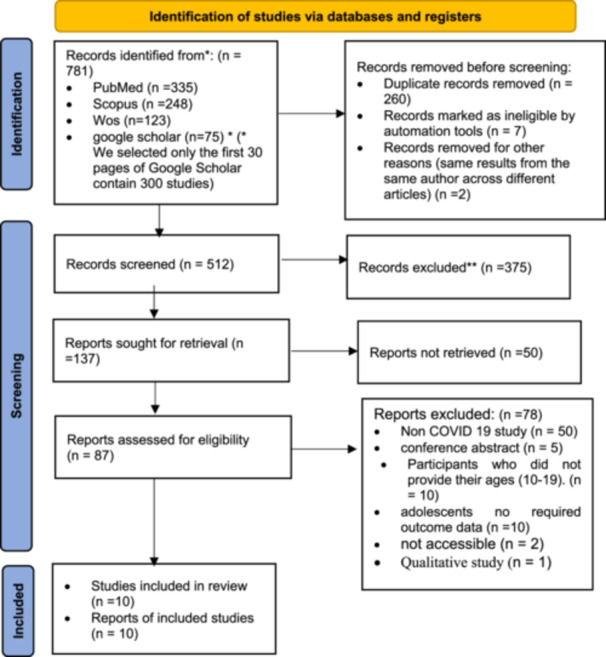
PRISMA flow diagram showing the selection process of the qualified articles.

The article selection process is depicted in Figure 1. After conducting a search using the specified keywords individually or in combination, 781 articles were identified following the removal of duplicates and a two‐level evaluation process. Subsequently, 10 studies were included in the systematic review. Due to the heterogeneity of the included data (see Figure 1).

### 3.2 Study Characteristics

The study involved a comprehensive sample of 636,873 participants. The analyzed articles were conducted between 2021 and 2024. In terms of design, the research included one cohort study [45], four cross‐sectional studies [46, 47, 48, 49], four RCT studies [41, 42, 44, 51], and one quasi‐experimental intervention study [43]. Geographically, the studies were distributed as follows: five in the United States, one in Canada, one in Thailand, one in Nepal, one in Italy, and one in Iran. The age of the participants ranged from 10 to 19 years. This review examines observational and interventional studies on adolescent sexual health during the COVID‐19 outbreak, focusing on SRH, prevention behaviors, sexual health literacy, knowledge of STIs, condom use, pre‐exposure prophylaxis (PrEP), HIV/STI testing, fear of contracting COVID‐19, sexual activity, contraceptive use, parental involvement, healthy and unhealthy romantic relationships, and knowledge of pornography. The results were evaluated using the 18‐item HIV Knowledge Questionnaire (HIV‐KQ‐18) [52], the 27‐item STD‐Knowledge Questionnaire (STD‐KQ) [53], and a self‐administered questionnaire from the Ministry of Public Health's Health Education Division [53].

Research has explored adolescent sexual health through various interventions, including a school‐based curriculum, media literacy approaches, the CARE tipping point program, and online HIV prevention initiatives. During the COVID‐19 era, these programs were implemented via web‐based platforms to provide sexual health education to adolescents aged 10−19, emphasizing group discussions. This review focused on RCTs where a control group received conventional treatment or usual care. The RCT was conducted using cluster‐randomized stepped wedge design (SWD) methods [38], with small randomized blocks and replacement blocks [41, 42, 43, 44] (see Table 1).

### 3.3 Risk of Bias of Included Studies

Out of the included studies, three RCTs were rated as having a low risk of bias, while one study were assessed as presenting some concerns regarding bias. Additionally, the risk of bias for one quasi‐experimental study, evaluated using the ROBINS‐1 tool, was rated as some concerns. Furthermore, several observational studies were included. The quality assessment of the studies revealed that five were rated as high quality (excellent) (refer to Table 1, and Appendix SB for detailed assessments).

The WHO defines sexual health as the enjoyment of sexual relations without exploitation, oppression, or abuse; safe pregnancy and childbirth; avoidance of unintended pregnancies; and prevention of STIs, including HIV. Improving access to, and information on sexual and reproductive service provision [54]. This systematic review examines the following variables based on the WHO definition: Five studies on SRH, two studies on knowledge of HIV/STIs, four studies on behaviors to prevent unintended pregnancies and STIs, and one study on dating violence and normative beliefs.

### 3.4 Synthesis of Results

The research on adolescent sexual health encompassed a range of factors as outlined earlier. However, due to the diversity and heterogeneity of the studies, a meta‐analysis of the sexual health outcomes was not feasible. Instead, a qualitative synthesis of the findings is provided below.

### 3.5 Main Findings

#### 3.5.1 Results From Observational Studies During the COVID‐19 Outbreak

##### 3.5.1.1 Reduced Access to SRH Services

Some studies reported declines in clinical SRH interactions (e.g., contraceptive counseling, STI testing) [45, 46, 49]. Also it was seen 28%–29% reduction in chlamydia/gonorrhea testing and a 19% reduction in syphilis/HIV testing. Due to this, chlamydia positivity rose from 10.4% to 12.7% (*p* = 0.003) and gonorrhea positivity doubled from 1.7% to 3.4% (*p* < 0.001) [47]. Vandermorris et al. [46] noted a 48% reduction in STI management visits in Canada, while Hong et al. [45] reported a 22% drop in HIV testing in the United States.

##### 3.5.1.2 Shift to Informal Information Sources

Italian adolescents relied heavily on the web and peers for SRH education, highlighting gaps in formal education systems [49].

##### 3.5.1.3 Exacerbated Health Inequities

Vulnerable groups (e.g., low‐income, rural, nonimmigrant adolescents in Canada; AMSM in the United States) experienced less improvement or greater service disruptions [45, 46].

##### 3.5.1.4 Pandemic Impact on Sexual Behavior

Italian adolescents reported strained relationships [49], while AMSM maintained stable condomless sex rates [45]. Canadian females had reduced pregnancies, potentially due to decreased partner access [46].

Policy gaps*:* Lack of sexual health education [48] and insufficient STI testing infrastructure highlighted systemic vulnerabilities [47].

#### 3.5.2 Results From Intervention Studies During the COVID‐19 Outbreak

##### 3.5.2.1 Access to SRH Services

US studies showed that access to these services increased sexual activity (IRR = 2.8), especially among those with fewer partners during COVID‐19 (IRR = 3.6).

Engaging in protected vaginal or anal sex significantly reduced negative outcomes (IRR = 0.25).

The intervention group had higher rates of protected sexual activity compared to the control group [40].

However, a quasi‐experimental study in Thailand found no significant differences in sexual health literacy, contraceptive use, or STI‐related behaviors between the experimental and control groups [43].

##### 3.5.2.2 Sexual Health Communication With Parents

Girls in the intervention group reported more frequent sexual health discussions with parents (M = 2.17) compared to the control group (M = 1.80). No significant changes were noted for boys.

Gender and condition had a significant impact on these communication patterns (*p* < 0.05) [41].

##### 3.5.2.3 Dating Violence

Acceptance of dating violence varied significantly by gender and intervention condition (*p* < 0.05). Boys in the intervention group showed lower acceptance of dating violence (M = 1.44), while girls did not exhibit significant changes [41].

##### 3.5.2.4 Normative Beliefs About Teen Sex

A normative belief about teen sex refers to shared cultural expectations or social norms regarding sexual behavior among adolescents. These norms shape how teens perceive and engage in sexual activities [55]. A significant gender‐condition interaction (*p* < 0.05) was observed.

Post‐intervention, girls in the experimental group perceived lower peer sexual activity (M = 32.06) compared to control group girls (M = 40.44; d = 0.38). No significant change was observed in boys (d = 0.01) [41].

##### 3.5.2.5 HIV/STI, Condom, and SRH Knowledge Scores

The intervention group showed higher median scores in HIV/STI awareness and knowledge post‐intervention and at a 3‐month follow‐up compared to the control group.

The intervention influenced views on pornography's impact on sexual behavior (OR 0.95) and ideal partner appearance (OR 1.12).

Both groups had similar median condom knowledge scores (5) post‐intervention with no significant differences [42].

In Nepal, the TPP+ group had significantly higher SRH knowledge scores and aspirations for education and marriage compared to the control group [44].

This summary encapsulates key findings from various intervention studies focusing on SRH during the COVID‐19 pandemic, highlighting improvements in communication, knowledge, and attitudes related to sexual health.

## 4 Discussion

In general, the analysis indicates that the implementation of interventional and observational studies aimed at assessing and improving adolescent sexual health has been limited during the COVID‐19 pandemic. Furthermore, a majority of the conducted studies lacked robust methodologies. The investigation's emphasis on various aspects of sexual health hindered its possibility of unification and meta‐analysis.

### 4.1 The Impact of COVID‐19 on Adolescent Sexual Behavior and Access to SRH Services

The studies in our review found a significant decline in HIV testing during COVID‐19, along with a slight drop in STI testing and PrEP usage. Fewer adolescents and young adults were tested for STIs in high‐prevalence areas, while chlamydia and gonorrhea positivity rates rose. Although adolescent pregnancy rates and the use of sexual health services were lower than expected, the decline in pregnancy rates among vulnerable adolescents was less pronounced. These data reveal that many young people have remained sexually active during the pandemic, despite social distancing recommendations, leaving them vulnerable to STIs. Rather than reducing STI risk, school shutdowns have been linked to stable or rising rates of infections [56, 57]. The COVID‐19 pandemic and the associated public health measures have significantly disrupted people's lives and their interactions with peers. These measures have also created additional barriers to accessing HIV prevention services, as many sexual health clinics reduced their operating hours or closed altogether in response to physical distancing orders. As a result, there has been decreased access to routine testing for HIV and other STIs, as well as to resources such as condoms, lubricants, and PrEP [58].

However, conflicting research indicated that studies reveal a decrease in sexual desire and the frequency of sexual activity during the COVID‐19 pandemic, primarily attributed to feelings of isolation and depression [59, 60]. Our study also revealed that the Covid‐19 pandemic has negatively affected relationships and sex lives, significantly impacting adolescents' health needs [48, 49].

### 4.2 SRH Intervention

The findings revealed a rise in sexual activity among adolescents who lacked access to healthcare services amidst the COVID‐19 outbreak. The sexual lives and activities of adolescents play a crucial role in establishing positive sexual relationships and expressing their sexuality [17]. However, an escalation in sexual activity without proper access to healthcare services can potentially result in difficulties obtaining condoms, HIV and STI testing, and treatment services, leading to an increase in rates of STIs and unintended pregnancies among adolescents [22, 61]. The current situation is concerning as the COVID‐19 pandemic has led to a decline in the availability of reproductive and sexual services. Consequently, it is reasonable to assume that the lack of access to these services may result in a rise in unsafe sexual activity, unintended pregnancies, and various associated issues [28].

### 4.3 Sexual Health Communication With a Parent

A review of the studies revealed that parents who educated their Adolescent daughters about sexual health issues had a beneficial effect on their daughters during the COVID‐19 pandemic. The COVID‐19 pandemic has significantly impacted adolescents' sexual lives, with social distancing and school closures reduced social interaction, while increased parental monitoring limited independence, peer interaction, and privacy [62]. The findings align with a study showing parents significantly shared reproductive health information with their children. The COVID‐19 pandemic improved parent−child communication, boosting children's awareness of sexual health [63]. Furthermore, adolescents often mimic their parents' behavior, making it crucial for parents to set a good example. However, challenges like school closures, remote work, economic strain, and pandemic‐related anxiety can hinder this. Thus, school nurses or school programs should take the lead in promoting effective adolescent health behaviors [64].

### 4.4 Dating Violence

The interventions reduced dating violence among boys but showed no significant impact on girls. These findings highlight the need for adolescent‐focused programs to address challenges from the COVID‐19 pandemic [65]. While effective for boys, the interventions failed to influence girls, raising questions about their adequacy in addressing factors like boys' condom use, multiple partnerships, and physical strength [66]. Future programs should challenge gender role beliefs and attitudes that justify violence, incorporating boys' perspectives on dating violence [67].

### 4.5 Normative Beliefs About Teen Sex

A community‐based program in Nepal aimed at advancing adolescent girls' rights reduced early and forced marriages before COVID‐19 by transforming societal norms and fostering girls' movements. During COVID‐19, the program only improved SRH knowledge, with no changes in other health indices despite disruptions and concurrent interventions [44]. The systematic review conducted by Meherali et al. reveals the profound impact of the COVID‐19 pandemic on adolescents' SRH, including restricted availability of SRH services, sexual or intimate partner violence, increased early marriages due to school closures, and disruptions in maternity care [28].

### 4.6 HIV/STI, Condom, and SRH Knowledge

The study found that sexual health literacy training during COVID‐19 did not significantly influence preventive behaviors for unwanted pregnancies and STIs. While health literacy affects fertility knowledge and outcomes, healthcare professionals must stay updated on best practices to ensure quality care, as inadequate training yields no behavioral improvements [68]. The educational programs implemented during the COVID‐19 pandemic proved to be successful in enhancing individuals' understanding of HIV and their perception of the influence of pornography on sexual behavior. The findings are consistently supported by numerous research studies [69, 70].

COVID‐19 measures had limited success in improving SRH behaviors, aside from raising AIDS awareness. Adolescent reproductive health was influenced by macrosystem factors like stress, poverty, quarantine, and weakened law enforcement, requiring government intervention [30]. Studies highlight concerns over rising teenage pregnancies during the pandemic [71, 72].

The global situation has revealed limited knowledge about interventions for adolescent sexual health during pandemics. Research in this area can help us better understand the impact of these interventions during the Covid‐19 pandemic. Our research centers on adolescent sexual health, with a particular focus on the challenges posed by the COVID‐19 pandemic. The outbreak has significantly disrupted adolescent sexual health, underscoring its urgency as a public health issue. To address this concern, we conducted our study during the pandemic, analyzing existing research on sexual health and exploring various intervention and educational programs. Our comprehensive evaluation examined both the pandemic's impact on adolescent sexual health and the effectiveness of implemented interventions. This study offers valuable insights and actionable data to support adolescents in maintaining their sexual health amid these unprecedented challenges.

## 5 Limitations

The current study has various limitations that should be considered. First, the documented consequences of the COVID‐19 pandemic on various outcomes are based on individuals' perceptions and are limited to a specific point in time. Second, the prevailing conditions of the epidemic have restricted face‐to‐face interventions and the comprehensive evaluation of their efficacy, particularly in small‐scale studies. Third, there is a scarcity of research on the sexual health of young individuals during the COVID‐19 outbreak.

Our study's limitations include nonuniform regions of sexual health, non‐identical sampling procedures, and the inclusion of only 10 studies, which prevented a meta‐analysis.

## 6 Conclusions

The COVID‐19 pandemic significantly disrupted adolescent SRH globally, reducing access to essential services and exacerbating existing inequities. While some behaviors such as sexual activity, demonstrated resilience, systemic gaps in education, and healthcare persist. Multisectoral efforts are needed to ensure adolescents' SRH rights are upheld during crises. However, the interventional studies underscore the viability of digital, media‐literate interventions in improving adolescent sexual health. This review compiles evidence on adolescent sexual health and highlights early interventions implemented during the pandemic, emphasizing both the negative impact of COVID‐19 on adolescent sexual health and the proven effectiveness of these interventions. Engaging adolescents in discussions about sensitive topics like sexuality has always been challenging. However, advancements in digital technologies have created opportunities for long‐lasting positive impacts beyond the pandemic. Future research should build on the groundbreaking programs identified in this analysis while addressing gaps in inclusivity. Enhancing adolescents' sexual health contributes positively to their overall sexual, mental, and physical well‐being throughout their lives. Addressing current challenges requires sustained efforts to uphold adolescents' rights, resilience, and access to essential resources for their growth and development.

## Author Contributions

**Elahe Ahmadnia:** conceptualization, methodology, supervision, validation, writing – review and editing. **Arezoo Haseli:** conceptualization, investigation, validation, writing, review – editing, supervision. **Atefeh Davoudian:** writing – original draft, investigation, resources, methodology. **Mina Abbasi:** writing – original draft, conceptualization, validation, methodology, resources, writing – review and editing.

## Conflicts of Interest

The authors declare no conflicts of interest.

## Transparency Statement

The lead author, Mina Abbasi, affirms that this manuscript is an honest, accurate, and transparent account of the study being reported; that no important aspects of the study have been omitted; and that any discrepancies from the study as planned (and, if relevant, registered) have been explained.

## Supporting information

Appendix A.

Appendix B.

## Data Availability

The data that support the findings of this study are available on request from the corresponding author. The data are not publicly available due to privacy or ethical restrictions.
