# Supplementary material for: Adolescents' Sexual Health During the COVID‐19 Outbreak: A Systematic Review
Source: Health Sci Rep. 2025 Apr 29;8(5):e70774. doi: 10.1002/hsr2.70774 (PMC12040736; doi:10.1002/hsr2.70774)
Supplement: Supplementary file 1 — Appendix A. [file HSR2-8-e70774-s001.docx]

**Appendix A: Search syntax**

PubMed (n =335)

(COVID-19[mh] OR COVID 19[tiab] OR 2019-nCoV Infection*[tiab] OR SARS-CoV-2 Infection*[tiab] OR Coronavirus Disease*[tiab] OR Coronavirus Infection*[tiab] OR COVID19[tiab] OR Coronavirus[tiab]) AND (Sexual Health[mh] OR (Sexual*[tiab] AND health*[tiab])) AND (Adolescent[mh] OR Adolescent*[tiab] OR Adolescence[tiab] OR Teenager*[tiab] OR Youth*[tiab])

WOS (n=123)

TS=((“COVID-19” OR “2019-nCoV Infection*” OR “SARS-CoV-2 Infection*" OR COVID19 OR Coronavirus) AND (“Sexual Health” OR (Sexual* NEAR/6 health*)) AND (Adolescent* OR Adolescence OR Teen* OR Teenager* OR Youth*))

Scopus (n =248)

TITLE-ABS-KEY((“COVID-19” OR “2019-nCoV Infection*” OR “SARS-CoV-2 Infection*" OR COVID19 OR Coronavirus) AND (“Sexual Health” OR (Sexual* W/6 health*)) AND (Adolescent* OR Adolescence OR Teen* OR Teenager* OR Youth*))

Google scholar (n=75) * (* We selected only the first 30 pages of Google Scholar contain 300 studies)

(“COVID-19” OR “2019-nCoV Infection*” OR “SARS-CoV-2 Infection*" OR COVID19 OR Coronavirus) AND (“Sexual Health”) AND (Adolescent* OR Adolescence OR Teen* OR Teenager* OR Youth*)
