# Supplementary material for: Adolescents' Sexual Health During the COVID‐19 Outbreak: A Systematic Review
Source: Health Sci Rep. 2025 Apr 29;8(5):e70774. doi: 10.1002/hsr2.70774 (PMC12040736; doi:10.1002/hsr2.70774)
Supplement: Supplementary file 2 — Appendix B. [file HSR2-8-e70774-s002.docx]

**Appendix B: Risk of bias of included studies**


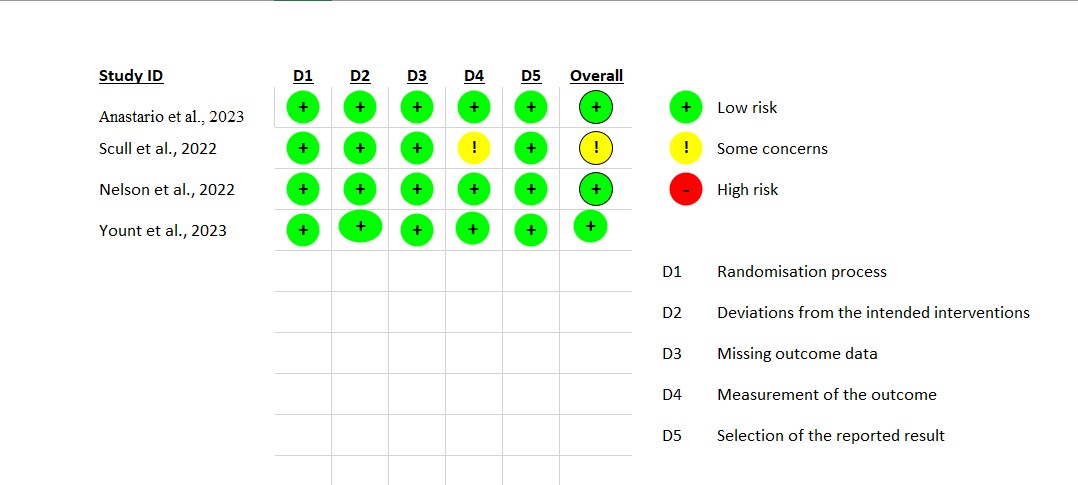


**Appendix B1**: Quality assessment of studies included in a systematic review using the Cochrane tool

**Appendix B2:** The Newcastle-Ottawa Scale (NOS) for assessing the quality of observational studies

| **Item Number**  **Author/Year** | **Study design** | **Selection** | | | | | | | | | | | | | | **Comparability** | | **Outcome/ Exposure** | | | | | | | | | | | **Total score** |
| --- | --- | --- | --- | --- | --- | --- | --- | --- | --- | --- | --- | --- | --- | --- | --- | --- | --- | --- | --- | --- | --- | --- | --- | --- | --- | --- | --- | --- | --- |
|  |  | **1** | | | | **2** | | | **3** | | | | **4** | | | **1** | | **1** | | | | | **2** | | **3** | | | |  |
|  |  | **a** | **b** | **c** | **d** | **a** | **b** | **c** | **a** | **b** | **c** | **d** | **a** | **b** | **c** | **a** | **b** | **a** | **b** | **c** | **d** | **e** | **a** | **b** | **a** | **b** | **c** | **d** | 7** |
| Hong, 2023, | Cohort | 1 | 0 | 0 | - | 1 | 0 | - | 1 | 0 | 0 | - | 1 | 0 | -* | 0 | 0 | 0 | 1 | 0 | 0 | 0 | 1 | 0 | 1 | 0 | 0 | 0 |  |
| Vandermorris, 2024 | Cross-sectional | 1 | 0 | 0 | 0 | 1 | 0 | 0 | 1 | 0 | 0 | - | 1 | 0 | 0 | 1 | 1 | 0 | 1 | 0 | 0 | - | 1 | 0 | - | - | - | - | 8 |
| Bonett, 2022 | Cross-sectional | 1 | 0 | 0 | 0 | 0 | 0 | - | 2 | 0 | 0 | - | 2 | 0 | 0 | 1 | 0 | 0 | 0 | 1 | 0 | - | 1 | 0 | - | - | - | - | 8 |
| Alamolhoda, 2021 | Cross-sectional | 1 | 0 | 0 | 0 | 1 | 0 | - | 2 | 0 | 0 | - | 2 | 0 | 0 | 1 | 0 | 1 | 0 | 0 | 0 | - | 1 | 0 | - | - | - | - | 9 |
| Montalti/ 2022 | Cross-sectional | 1 | 0 | 0 | 0 | 1 | 0 | - | 2 | 0 | 0 | - | 1 | 0 | 0 | 1 | 0 | 1 | 0 | 0 | 0 | - | 0 | 0 | - | - | - | - | 7 |

* -, No items; The NOS checklist included 3 items (multi-component). The scoring range of the NOS checklist was from 0 to 9. **High-quality articles with a score of 7 or higher are considered high-quality, medium-quality articles between 5 and 6, and low-quality articles with a score of 4 or less. 4 high-quality studies (excellent) were ranked.
